# Honey can repairing damage of liver tissue due to protein energy malnutrition through induction of endogenous stem cells

**DOI:** 10.14202/vetworld.2017.711-715

**Published:** 2017-06-29

**Authors:** R. Heru Prasetyo, Eka Pramyrtha Hestianah

**Affiliations:** 1 Department of Parasitology, Faculty of Medicine, Universitas Airlangga, Surabaya, East Java, Indonesia; 2 Department of Veterinary Anatomy, Faculty of Veterinary Medicine, Universitas Airlangga, Surabaya, East Java, Indonesia

**Keywords:** endogenous stem cells, honey, liver tissue, protein energy malnutrition, regeneration

## Abstract

**Aim:**

This study was to evaluate effect of honey in repairing damage of liver tissue due to energy protein malnutrition and in mobilization of endogenous stem cells.

**Materials and Methods:**

Male mice model of degenerative liver was obtained through food fasting but still have drinking water for 5 days. It caused energy protein malnutrition and damage of liver tissue. The administration of 50% (v/v) honey was performed for 10 consecutive days, while the positive control group was fasted and not given honey and the negative control not fasted and without honey. Observations of regeneration the liver tissue based on histologically examination, observation of Hsp70 expression, and homing signal based on vascular endothelial growth factor-1 (VEGF-1) expression using immunohistochemistry technique. Observation on expression of CD34 and CD45 as the marker of auto mobilization of hematopoietic stem cells using flow cytometry technique.

**Results:**

There is regeneration of the liver tissue due to protein energy malnutrition, decrease of Hsp70 expression, increase of VEGF-1 expression, and high expression of CD34 and CD45.

**Conclusion:**

Honey can improve the liver tissue based on: (1) Mobilization of endogenous stem cells (CD34 and CD45); (2) Hsp70 and VEGF-1 expressions as regeneration marker of improvement, and (3) regeneration histologically of liver tissue

## Introduction

Nutrition deficiency was an health problem, especially in development countries, include Indonesia. Since 2000 in Indonesia showed increased incidence of protein energy malnutrition (PEM) [1]. FAO mention that until 2016, more than 20 million Indonesian population is still undernourished [2]. More than 50% of deaths of malnourished children due to lack of energy protein which increases the number of deaths due to intestinal disorders and diarrhea [1].

In general, PEM is the most frequent cause of acquired immune deficiency [3] which can lead to opportunistic infection by intestinal parasites with symptoms of chronic diarrhea and that will increase mortality [4]. From previous studies, the mice were conditioned PEM in addition to obtained an increased expression of Hsp70, decreased expression of prostaglandin E2, and expression of immunoglobulin A were examined immunohistochemically (IHC), also histologically visible damage to the epithelium of the intestinal mucosa and occurs villus atrophy, which will lead to a decrease in the function of absorption [5] the impact reduced nutritional intake and organs damage, including liver damage.

This study aimed to evaluate the impact of honey to repair liver tissue and its effect on mobilization of endogenous stem cells in the liver tissue damage due to PEM. The results of this study are expected as input in determining policies for handling malnourished.

## Materials and Methods

### Ethical Approval

The present study was approved by ethical committee vide Ethical Clearance No: 065-KE (Komisi Etik Penelitian, Fakultas Kedokteran Hewan, Universitas Airlangga, Animal Care and Use Committee (ACUC)).

### PEM modeling causes liver degeneration

This study was started by PEM as the causes of liver degeneration of male mice. PEM was done by standing water every 8 hours for the male mice without any food in the course of 5 days [6,7]. Fasting was done in 5 days because in the 5^th^ day the liver undergoes degeneration and atrophy of the intestinal epithelial has been damaged [8]. This study used male Balb/c mice 8-10 weeks old, 20-30 g body weight in good condition [9].

Mice obtained from laboratory animals experiments in Faculty of Veterinary Medicine, Universitas Airlangga. Mice kept in a plastic cage space per individual in laboratory animals experiments in Faculty of Veterinary Medicine, Universitas Airlangga.

### Research Design

The research is an experimental study with design the randomized post-test only control group design. The study was divided into 4 groups of 10 mices each (based formula (t-1)(n-1)>15). t (treatments)=4 groups and n (replicates)=6 (minimal). Description of treatment:

1. The negative control group (T1): Male mices not fasted and without honey.
2. The positive control group (T2): Male mices were fasted for 5 days and without honey.
3. The treatment group (T3): Male mices not fasted for 5 days, then given 50% (v/v) honey in the drinking water for the next 5 days after fasted.
4. The treatment group 4 (T4): Male mices were fasted, then given 50% (v/v) honey in the drinking water for the next 5 days after fasted.

Honey that used in this study was raw multiflora honey, *Apis dorsata* product from Batu Malang, East Java, Indonesia.

After the treatment, whole blood is taken from the heart and is collected in heparin tube, then performed euthanasia by cervical dislocation and follow by making the liver organ. The liver organ put in a pot that already contains 10% formalin solution after cleansing with saline.

Observations were taken for regeneration of liver tissue used histologically technique, Hsp70 expression and vascular endothelial growth factor-1 (VEGF-1) expression used IHC technique, and the percentage of CD34 and CD45 used flow cytometry technique.

### Observation of hematopoietic stem cells mobilization as endogenous stem cells based on expression of CD34 and CD45 with flow cytometry method

After the mice were treated, further examination of whole blood sample is taken through cardiac puncture and inserted into the tube was content heparin to prevent coagulation. Observations on the expression of CD34 and CD45 were analyzed by flow cytometry

Flow cytometry method, starting with the preparation of whole blood centrifugation in a temperature of 4°C, with a speed of 6000 rpm for 15 min. Results centrifuging the cell in the form of sludge mixed with cytoperm amount of 2 times the number of cells are analyzed. A mixture of cells and cytoperm was centrifuged to obtain a supernatant and a pellet. Washing the pellet amount 4 times of obtained cell number from the first centrifugation.

Furthermore, add lysis buffer amount of 2 times of the first obtained cell number. After that add labeled antibody conjugate to each sample, five tubes are prepared and processed in parallel. (1) Single staining with CD34 PE added to the wash tube. (2) Double staining with CD34 PE and CD45 PerCP and CD105 FITC wash tube. (3) Double staining with CD34 PE and CD45 PerCP trucount tube. The entire sample was then stored at 4°C in the dark and analyzed using flow cytometry for 1 h [10].

### IHC technique for observation of Hsp70 and VEGF-1 expression

IHC observation was performed to determine the expression of Hsp70 as a marker of improvement and VEGF-1 as a marker of growth factor. Before to IHC methods were made histological preparation, by way of an incision is made transversely liver tissue from paraffin blocks. Further examination by making outward through IHC techniques using monoclonal antibodies anti-Hsp70 and monoclonal antibodies anti VEGF-1. Observations of Hsp70 and VEGF-1 expression were made using a light microscope with a magnification of 200 times, and the expression of each variable is indicated by the number of cells with brownish discoloration chromogen in each incision [11].

### Histologically technique

Histologically examination based on observation of liver tissue regeneration. Histologically examination begins with the making of histological preparations, such as the following: Mice liver fixation in 10% buffer formalin. Subsequently, liver dehydrated in alcohol solution with a higher concentration, i.e., from 70%, 80%, 90%, and 96% (absolute). Then do the clearing in the liver of mice in xylol solution. Furthermore, performed embedding using liquid paraffin and mice liver were put into molds containing liquid paraffin. Before stained and sectioning performed, an incision using a microtome and mounted on glass objects. Furthermore is done the staining by removing of paraffin with xylol then put into a solution of alcohol with decreased concentration and then put into stain matter. The last stage after stained is done mounting, put into water or alcohol to remove excess stain. Then put into a solution of alcohol with increasing concentration, and then put into xylol. Preparations then covered with a cover glass and mounted with entellan. Microscopic examination is done with a light microscope with a magnification 200× [12,13].

### Statistical analysis

Expressions of mice liver, VEGF-1, and Hsp70 were statistically analyzed using SPSS 15 for Windows XP with the level of significance 0.05 (p=0.05) and the confidence level 99% (α=0.01). Steps of comparative hypothesis tests are as follows: Test data normality with the Kolmogorov–Smirnov test, homogeneity of variance test, analysis of variance factorial, *post hoc* test (least significant difference test) using the Tukey highly significant difference 5%.

## Results and Discussion

Data were collected from 32 male mices were divided into four treatments: Negative control group (T1) is normal liver without honey; positive control group (T2) is degenerative liver without honey; (T3) group is normal liver + 50% (v/v) honey in drinking water for 5 days; (T4) group is degenerative liver + 50% (v/v) honey in drinking water for 5 days. In detail, the results of the study are as follows: The effectively of honey was based on: (1) mobilization of endogenous stem cells (CD34 and CD45); (2) Hsp70 and VEGF-1 expressions as regeneration Hsp70 as a marker of improvement [8] and VEGF-1 as a marker of growth factor [14]; and (3) regeneration histologically of liver tissue explain, why HSP70 and VEGF-1 are checked as markers.

**Figure-1:**
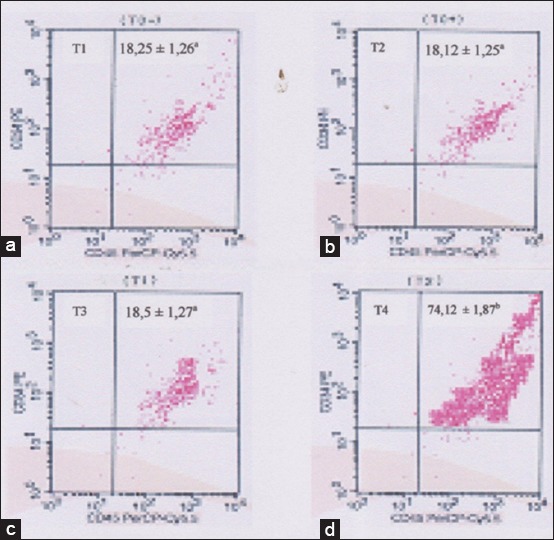
Flow cytometry analysis of endogenous stem cells mobilization. (a) The control negative group (T1): Expression of CD34 and CD45 of 18.25±1.26^a^. (b) The control positive group (T2): Expression of CD34 and CD45 of 18.12±1.25^a^. (c) The group 3 (T3): Expression of CD34 and CD45 of 18.5±1.27^a^. (d) The group 4 (T4): expression of CD34 and CD45 of 74.12±1.87^b^. The different superscripts indicate significant difference at p<0.05.

Mobilization of endogenous stem cells analyzed by flow cytometry based on increased concentration of CD34 and CD45. The analysis shows that: Either a negative control group (T1), positive control group (T2) or group (T3) showed no mobilization of endogenous stem cells, based on the percentage of CD34 and CD45 which are at a percentage of <20% (Figure-1a-c), whereas in the group (T4) showed mobilization of endogenous stem cells is based on the percentage of CD34 and CD45 which are located on the percentage of over 70% (Figure-1d). Based on statistical calculations T2 groups was significantly different (p<0.05) than the other three treatments (T1, T2, and T3), whereas among the three treatments no significant difference (p>0.05) (Figure-1). The process of mobilization can occur in several ways, among others are the immune responses or inflammatory reaction due to injury signals (cytokines, nuclear factor kappa B, β catenin through Wnt) from the tissue damage and homing signals like VEGF-1 that appears if there are an inflammatory reaction. Homing signal that occurs plays a role in the recruitment of stem cells as well as in cells undergoing apoptosis [15-17].

**Figure-2:**
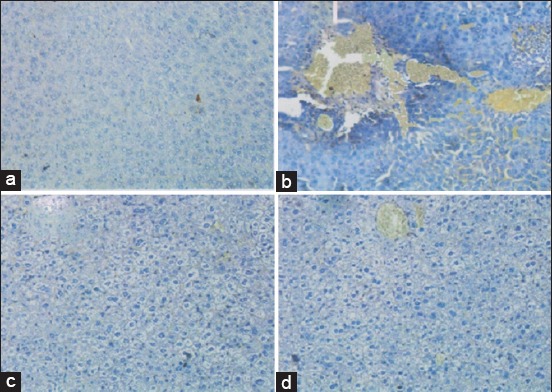
Hsp70 Expression in mice liver tissue using immunohistochemically technique on several treatments. (a) The control negative group (T1), score Hsp70 expression=0.125±−0.90^a^; (b) The group 3 (T3), score Hsp70 expression=1.25±−0.09^b^. (c) The control positive group (T2), score Hsp70 expression=0.375±−0.43^a^. (d) The group 4 (T4), score Hsp70 expression=2.875±0.46^c^. The different superscripts indicate significant difference at p<0.05.

Hsp70 expression in this study showed a decrease in the group receiving honey, either in group T3 (0.375±−0.43^a^) and T4 group (1.25±−0.09^b^). Decreased expression of Hsp70 after the addition of honey on T3 groups was not significant (p>0.05) with T1 group (0.125±−0.90^a^). In the T4 group despite the downturn but significantly differ significantly with T1 and T3 groups (p<0.05). This indicates that the honey treatment, on a molecular basis is not optimal. In the T2 group (2.875±0.46^c^) an increase in excessive and significantly different when compared with the other 3 groups. This shows liver tissue damage is experiencing PEM and not be given honey (Figure-2). Decrease of Hsp70 expression indicates apoptosis inhibition and regeneration of liver tissue [18].

**Figure-3:**
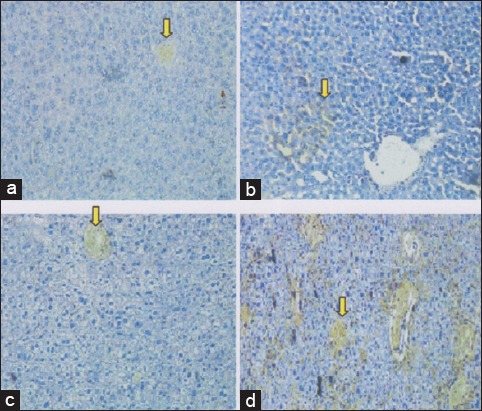
Vascular endothelial growth factor-1 (VEGF-1) expression in mice liver tissue using immunohistochemically technique on several treatments. (a) The control negative group (T1), score VEGF-1 expression=0.25±−0.60^a^; (b) The group 3 (T3), score VEGF-1 expression=0.375±−0.43^a^. (c) The control positive group (T2), score VEGF-1 expression=1.125±−0.78^b^. (d) The group 4 (T4), score VEGF-1 expression=2.75 ±0.44^c^. The different superscripts indicate significant difference at p<0.05.

The increased of growth factor based on VEGF-1 expression, in the normal control group (T1) was on the score 0.25±−0.60^a^ (VEGF-1 expression between 1% and 5%). The group of liver degenerative (T2) was on the score 1.125±−0.78^b^ (VEGF-1 expression between 6% and 25%). The group use 50% (v/v) bee-honey (T4) was on the score 2.75±0.44^c^ (VEGF expression >50%) (Figure-3). This shows an improvement of liver tissue through a process of homing signals [19].

**Figure-4:**
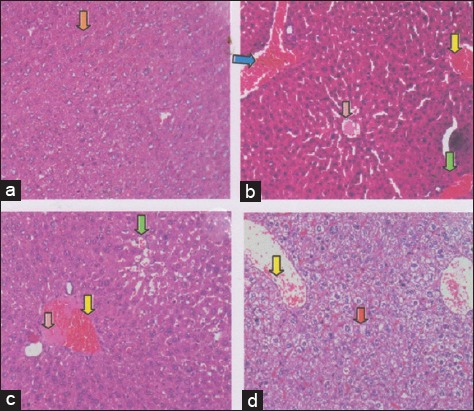
The regeneration of mice liver tissue using the histologically technique with hematoxylin and eosin staining in a few treatments. (a) The control negative group (T1) was showed normal hepatocytes expression (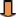); have abundant clear cytoplasm which has a “feathery” appearance due to the presence of glycogen. (b). The control positive group (T2), congestion of liver (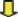) and hemorrhagi (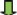), also visible hemosiderin (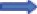) due to blood cell lysis (brownish yellow color) with deposition of fibrin (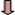) indicating that chronic congestive has occurred. Hepatocytes with condensed darkly staining cytoplasm because depleted of glycogen. (c). The group 3 (T3), liver does not regenerate, it appears there is congestion (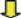) and deposition of fibrin (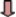). (d) The group 4 (T4), liver begin to regenerate with hepatocytes expression which production of both microvesicular and macrovesicular vacuolation (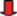), although it still looks little congestion in blood vessel (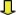).

Furthermore, effectiveness of honey in this study based on the regeneration of the liver. The regeneration can be observed through the method of histopathology anatomy with hematoxylin and eosin (H and E) staining. Microscopic examination showed that the group of 50% (v/v) honey (T4), leading to the occurrence of liver tissue repair, based on hepatocytes expression and production of both microvesicular and macrovesicular vacuolation (Figure-4d). The control negative group (T1) with normal liver has normal hepatocytes expression, have abundant clear cytoplasm which has a “feathery” appearance due to the presence of glycogen (Figure-4a). The control positive group (T2), congestion of liver and hemorrhage, also visible hemosiderin due to blood cell lysis (brownish yellow color) with deposition of fibrin indicating that chronic congestive has occurred. Hepatocytes with condensed darkly staining cytoplasm because depleted of glycogen (Figure-4b) Not the improvement in the form of liver does not regenerate, it appears there is congestion, hemorrhagi and hemosiderin expression (Figure-4c). The group of liver degenerative (T1), liver was congested extensive and hemorrhagi, also visible hemosiderin (yellow-brown) due to blood cell lysis with fibrin deposition (baby color pink) indicating that chronic congestion has occurred (Figure-4d).

These results indicate that administration of honey with a dose of 50% (v/v) in drinking water for 5 days was able to induce endogenous stem cells to migrate to where the defect. Repair begins on intestine organ, making it easier for the absorption of honey consumed. This ultimately led to the process of repair of liver tissue. Improvements to the liver can be seen in the results of histopathological examination of the anatomy of the liver tissue with H and E staining.

PEM in this study also caused fibrosis in liver tissue were experiencing defect. According to induced stem cells have the ability as antifibrotic so that honey therapy can be used in the treatment of tissue fibrosis related to the chronic inflammatory condition due to PEM [20,21].

Furthermore, trigger of VEGF-1 which binding to VEGF receptor-1 (VEGFR-1) can occurred. VEGF-1 is a homodimeric glycoproteins with a molecular weight of 45 kDa. VEGF-1 is expressed by many types of cells as homing signal. VEGF is a component of the extracellular matric from stem cells has a role in supporting a conducive microenvironment for stem cells. Trigger presence of VEGF-1 - VEGFR-1 will pass a series of signaling that activates stem cells factor (SCF) interstitial. SCF or also known as steel factor is a signaling protein mechanic on a niche so that physiologically communication will occur [22,23].

Treatment of 50% (v/v) honey for 5 days in male mice with degenerated liver tissue can show: (1) Mobilization of endogenous stem cells, in the form of markers CD34 and CD45 are expressed in blood serum; (2) induce homing signal in the form of increase of growth factor (VEGF-1) by IHC in liver tissue; (3) regeneration of liver tissue, hepatocytes expression and production of both microvesicular and macrovesicular vacuolation, although there is still little congestion but hemosiderin expression and fibrin deposition has not looked back. The results show that the wound healing properties of honey include stimulation of tissue growth and enhanced epithelialization. These effects are ascribed to nutritional and antioxidant contents and stimulation of immunity [24-26].

## Conclusion

Honey can be improvement the liver tissue based on: (1) Mobilization of endogenous stem cells (CD34 and CD45); (2) Hsp70 and VEGF-1 expressions as regeneration marker of improvement, and (3) regeneration histologically of liver tissue.

## Authors’ Contributions

RHP: Research coordinator, prepared PEM modeling causes liver degeneration in male mice, and administration of 50% (v/v) honey in the drinking water for the next 5 days after fasted and revised the manuscript. EPH: Method of histopathology anatomy and statistical analysis. All authors read and approved the final manuscript.
